# Phase-Separated Structure of NBR/PVC Blends with Different Acrylonitrile Contents Investigated Using STEM–EDS Mapping Analysis

**DOI:** 10.3390/polym15163343

**Published:** 2023-08-09

**Authors:** Yuka Komori, Aoi Taniguchi, Haruhisa Shibata, Shinya Goto, Hiromu Saito

**Affiliations:** 1 Materials Engineering R & D Division, DENSO CORPORATION, Kariya-shi 448-8661, Aichi, Japan; 2 Department of Organic and Polymer Materials Chemistry, Tokyo University of Agriculture and Technology, Koganei-shi 184-8588, Tokyo, Japan

**Keywords:** concentration distribution, DMA, NBR, phase-separated structure, polymer blend, PVC, STEM–EDS

## Abstract

We investigated the phase-separated structure of nitrile butadiene rubber (NBR)/polyvinyl chloride (PVC) blends with different acrylonitrile (AN) contents in the NBR, using dynamic mechanical analysis measurements and scanning-transmission-electron-microscopy (STEM)–energy-dispersive-X-ray-spectroscopy (EDS) elemental analysis. Two separate sharp tan *δ* peaks were observed in the blend at the lower AN content of 18.0%, whereas a broad peak was observed in the blends with the higher AN contents of 29.0 and 33.5%, due to the increase in miscibility, as expected from the decrease in the solubility parameter difference with the increasing AN content. The STEM–EDS elemental analysis for the concentration distribution showed that the NBR was mixed in the large PVC domains with a diameter of several micrometers, and the excluded PVC existed around the interface of the domain–matrix phases in the blend with the lower AN content, whereas small domains with a diameter of several tens of nanometers were dispersed in the blend with the higher AN content. The concentration difference in PVC between the PVC domain and the NBR matrix became smaller with increasing miscibility as the AN content increased although the blends contained the same PVC content of 40 wt%.

## 1. Introduction

A polymer blend is a mixture of chemically different polymers. Dissimilar polymers are only miscible via a favorable specific interaction between them, and most pairs of dissimilar polymers are phase-separated [1,2,3,4]. The degree of miscibility has been estimated from the glass transition, depending on the blend composition, using differential scanning calorimetry and dynamic mechanical analysis (DMA) [5,6,7]. A single glass transition is seen at intermediate temperatures between those of the component polymers in miscible blends, whereas two separate glass transitions are seen in phase-separated blends. A shift in the glass transition temperature is observed in the partially miscible blends, in which the component polymers are mixed at the interface of the two phases [8,9,10,11,12], or each component polymer exists in the phase of the partner polymers, due to the liquid–liquid phase separation in the two-phase region of the phase diagram [13]. Such a structure difference is crucial for tailoring the properties of polymer blends to practical applications. For instance, a broad tan *δ* peak is desirable for the application of damping materials [14,15,16,17,18]. To understand the details of the structure of polymer blends, depending on the degree of miscibility, the direct observation of the concentration distribution of the phase-separated structure is required. Scanning-transmission-electron-microscopy (STEM)–energy-dispersive-X-ray-spectroscopy (EDS) elemental analysis is considered to be useful for the observation of the phase-separated structure. However, the concentration distribution of the phase-separated structure has not been obtained via STEM–EDS elemental analysis using a single lithium–silicon detector [19]. A recent multidetector system for EDS using multiple silicon drift detectors (SDDs) enables elemental maps to be obtained, with a high detection efficiency [20,21,22].

To understand the possibilities of STEM–EDS elemental mapping analysis for polymer blends, the NBR/PVC blend can be used as a model system, because PVC contains the chlorine element, which can be used as the target element of the STEM-EDS elemental mapping analysis for the concentration distribution. The blend of NBR and PVC has been studied extensively, because it is applied on automotive parts, cable insulation, and wires, due to its excellent processability and elasticity, and its resistance to chemicals and the weather [23,24,25,26,27,28,29]. The NBR/PVC blends are known to be miscible at wide-blend compositions when the acrylonitrile (AN) content of the NBR is 23–45% [30,31,32]. On the other hand, a two-phase structure has also been observed in the blends in the same AN content range of 23–45% [19,33,34]. Recently, we found that the NBR/PVC blends with an AN content of 29.0% exhibited a lower critical solution temperature-type phase behavior, in which a miscible blend tends to phase-separate with elevating temperature [13]. Hence, the miscibility and phase-separated structure are considered to be changed depending on the AN content of the NBR at the same blend composition. However, the variation in the miscibility and the related structure depending on the AN content has not been clarified, despite the extensive studies on NBR/PVC blends.

In this paper, to clarify the variation in the degree of miscibility and the related structure in NBR/PVC blends with different AN contents in the NBR, we prepared a series of NBR/PVC blends with the different AN contents of 18.0%, 29.0%, and 33.5%. The degree of miscibility was evaluated based on the change in the glass transition of NBR and PVC, using DMA measurements, and the solubility parameter difference in the NBR and PVC. To clarify the detail of the phase-separated structure, we carried out direct observation of the concentration distribution of PVC in the NBR/PVC blends, via STEM–EDS elemental mapping analysis, with chlorine as the target element for the PVC. The concentration distribution of phase-separated structure depending on the AN content is discussed, in terms of the degree of miscibility.

## 2. Materials and Methods

### 2.1. Materials

Three kinds of NBR with different AN contents were supplied by Zeon Corp., Tokyo, Japan; i.e., grades DN401, 1043, and DN212. The AN contents of DN401, 1043, and DN212 were 18.0%, 29.0%, and 33.5%, respectively. The low content of AN (18.0%), medium content of AN (29.0%), and high content of AN (33.5%) are abbreviated as NBR-L, NBR-M, and NBR-H, respectively. Here, the Mooney viscosity obtained from the supplier [ML (1 + 4) 100 °C] for all three types of NBR was 77.5, suggesting that the molecular weight of the three kinds of NBR is the same. The PVC was supplied by Taiyo Vinyl Corp., Tokyo, Japan, grade TH-1300. 

ADK STAB SC-308E was used as the stabilizer of the PVC. Colloidal sulfur was used as the cross-linking agent of the NBR. Zinc oxide (ZnO), stearic acid, *N*-cyclohexyl-2-benzothiazolylsulfenamide (CZ), and tetramethylthiuram disulfide (TT) were used as the vulcanization accelerators. The grade, supplier, and additive amount of the stabilizer and additives are listed in Table 1.

### 2.2. Sample Preparation

The blend specimen was prepared using a melt-mixing method. The NBR and PVC were melt-mixed at a rotor speed of 200 rpm at 150 °C for 10 min, in a mixing chamber of a miniature mixing machine (IMC-18D7, Imoto Machinery Co., Ltd., Kyoto, Japan). The composition of the NBR/PVC blend was fixed at 60/40. To prevent a chemical reaction between the NBR and PVC, the stabilizer was added during the mixing. Then, the cross-linking agent of sulfur, and the vulcanization accelerators of ZnO, stearic acid, CZ, and TT were added, and were mixed at 100 °C for 10 min. To obtain the blend film specimen with a thickness of 400 μm and to cross-link the NBR in the blend, the specimen thus obtained was compression-molded between two flat aluminum plates at 150 °C for 30 min, using a hot press machine (Imoto Machinery Co., Ltd., Kyoto, Japan).

### 2.3. DMA Measurement

The DMA measurement was carried out using a dynamic mechanical analyzer (RSA G2, TA Instruments Inc., New Castle, DE, USA). The dimensions of the specimen were 3 mm wide, 0.4 mm thick, and 30 mm long, with a gap distance of 5 mm. The specimen was heated from −60 to 150 °C, at a heating rate of 5 °C/min, and at a constant oscillatory frequency of 10 Hz, in the tensile mode. The storage modulus *E′* and loss modulus *E*″ were obtained as a function of the temperature, and the loss tangent (tan *δ*) was given by tan *δ* = *E*″/*E*′.

### 2.4. STEM Observation

The phase-separated structures of the blends were observed via STEM, using a TEM instrument (JEM-F200, JEOL Co., Ltd., Tokyo, Japan). The STEM image was acquired in bright-field (BF) mode, at an acceleration voltage of 200 kV. The film specimen was cut to an ultrathin section of 100–150 nm thickness, using a microtome at −100 °C, and the ultrathin section was stained with RuO_4_ under a vacuum at 20 °C. The sections were then placed on a microgrid copper mesh (780111613, JEOL Co., Ltd.). The distribution of the component polymer of the PVC in the observed area was obtained via elemental mapping analysis for chlorine, using EDS equipped with dual SDDs (EX-24390UBN5T dry SD100WL, JEOL Co., Ltd.), attached in both parallel and vertical directions to the sample holder of the TEM instrument. EDS elemental mapping was carried out using an electron probe with 0.18 nm diameter and 0.2 nA current, and the elemental maps of 512 × 512 pixels were obtained by scanning the 2D region, and the accumulation of the EDS spectra for 13.3 s for each frame of 512 × 512 pixels. All the elemental maps used a pixel dwell time of 50 μs, with averaging of at least 25 frames, with drift compensation. The maps and the extracted EDS spectra were quantified using the Cliff–Lorimer method [35], with Pathfinder X-ray Microanalysis software (Thermo Fisher Science Co., Ltd., Waltham, MA, USA). Note here that carbon (C), nitrogen (N), and chlorine (Cl) were selected from the elements detected in the EDS spectra, and the peak intensities of the C, N, and Cl spectra were obtained as *I*_C_, *I*_N_, and *I*_Cl_, respectively. The atomic concentration in atm% for the Cl element of PVC was estimated as a relative value by *I*_Cl_*k*_Cl_/(*I*_C_*k*_C_ + *I*_N_*k*_N_ + *I*_Cl_*k*_Cl_), using the Cliff–Lorimer *k*-factors of each element, *k*_C_, *k*_N_, and *k*_Cl_ [35].

## 3. Results and Discussion

### 3.1. Glass Transition

**Figure 1:**
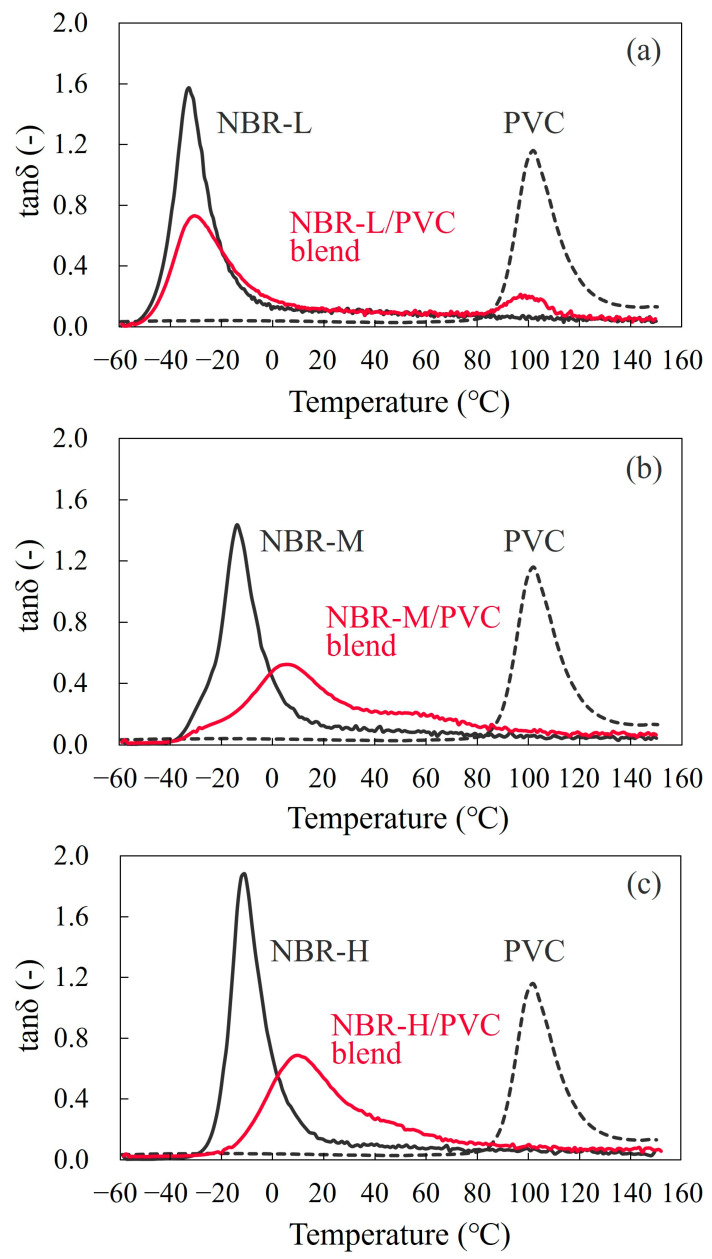
Tan *δ* at a heating rate of 5 °C/min for the 60/40 NBR/PVC blends with a different AN content in the NBR, and the neat component polymers: (**a**) NBR-L/PVC; (**b**) NBR-M/PVC; (**c**) NBR-H/PVC.

Figure 1 shows the loss tangent (tan *δ*) with temperature obtained via DMA measurement for the 60/40 NBR/PVC blends with different AN contents in the NBR; i.e., NBR-L (low AN content of 18.0%), NBR-M (medium AN content of 29.0%), and NBR-H (high AN content of 33.5%). The tan *δ* with temperature for the neat NBR and PVC is also shown in Figure 1 for comparison. The tan *δ* peaks observed at approximately −33 °C and 100 °C were derived from the glass transitions of the neat NBR-L and neat PVC, respectively. Two separate sharp tan *δ* peaks were seen in the NBR-L/PVC blend (Figure 1a). One peak that emerged at approximately −30 °C was derived from the glass transition of the NBR-L phase, whereas another peak that emerged at approximately 97 °C was derived from the glass transition of the PVC phase, due to the existence of the phase-separated structure of the NBR-L and PVC. A small shift to a lower temperature was seen in the high-temperature peak for the glass transition of PVC, and a small tail to higher temperature was seen in the low-temperature peak for the glass transition of NBR-L. The small shift and small tail in the two separate tan *δ* peaks are usually considered to be attributed to the miscibility of the component polymers at the interface [8,9,10,11,12].

Two separate broad tan *δ* peaks were seen in the NBR-M/PVC blend, and they were greatly shifted from the glass transitions of the NBR-M and PVC (Figure 1b). The tan *δ* peak derived from the glass transition of the neat NBR-M observed at −14 °C was shifted to 6 °C, whereas that of the PVC observed at 100 °C was shifted to 56 °C. This indicates that the glass transition of the NBR-M was shifted 20 °C higher in the blend, whereas that of the PVC was shifted 44 °C lower in the blend. The tan *δ* peaks of the neat component polymers became broader in the wide temperature region in the blend. The large shift and broadness of the glass transitions in the blend are attributed to the partial miscibility of the NBR-M and PVC in the two-phase structure of the NBR-M/PVC blend.

A single broad tan *δ* peak was seen in the NBR-H/PVC blend (Figure 1c). The tan *δ* peaks derived from the glass transition of the neat NBR-H observed at −11 °C, and that of the neat PVC observed at 100 °C were merged to a single peak at 10 °C in the blend. The tan *δ* peak was asymmetric, and a wide tail was seen at the high-temperature region of 35–60 °C in the NBR-H/PVC blend. A blend with a single tan *δ* peak is usually considered to be a single-phase miscible one, although the peak is asymmetric, and has a wide tail.

Thus, a series of NBR/PVC blends with different degrees of miscibility and phase-separated structure were obtained, depending on the AN content in the NBR at the same blend composition of 60/40; i.e., the miscibility was highest in the NBR-H/PVC blend, followed by the NBR-M/PVC blend, and it was lowest in the NBR-L/PVC blend.

### 3.2. Solubility Parameter

The miscibility of the component polymers in polymer blends can be evaluated using the Flory–Huggins interaction parameter of component polymers 1 and 2, *χ*_12_. The value of *χ*_12_ is given by:

(1) $$\chi_{12}=V_{0}{\left(\delta_{1}-\delta_{2}\right)}^{2}/RT$$

where *V*_0_ is the molar volume of the segment; *δ*_1_ and *δ*_2_ are the solubility parameters of component polymers 1 and 2, respectively; *R* is the gas constant; and *T* is the absolute temperature. Because *χ*_12_ decreases as the miscibility increases, the solubility parameter difference between the component polymers 1 and 2, Δ*δ* = *δ*_1_ − *δ*_2_, is a measure of miscibility; i.e., Δ*δ* is smaller as the degree of miscibility increases.

The Hildebrand solubility parameter of polymer i, *δ_i_*, is described by [36,37]:

(2) $$\delta_{i}=\sqrt{E_{C}/V}$$

where *E_C_* is the cohesive energy of the polymer, and *V* is its molar volume. The *E*_C_ and *V* of the NBR and PVC were calculated by the sum of contributions from their constituents, and the *δ*_i_ was calculated using Equation (2) from the *E*_C_ and *V* thus obtained, using Fedor’s method [36]. Table 2 shows the calculated *E*_C_, *V*, and *δ*_i_ of the NBR and PVC, and the solubility parameter difference Δ*δ* (=*δ*_PVC_ − *δ*_NBR_). The calculated procedure of the solubility parameter and the *E*_C_ and *V* of the constituents of the NBR and PVC used in the calculation are shown in Figure S1, as Supplementary Material. The solubility parameter of the NBR varied depending on the AN content. The solubility parameter difference, Δ*δ*, became smaller in the order of NBR-L/PVC blend, then NBR-M/PVC blend, then NBR-H/PVC blend; i.e., the miscibility is considered to increase with an increase in the AN content at the AN contents of 18.0%, 29.0%, and 33.5%. Thus, the difference in the miscibility indicated by the DMA results shown in Figure 1 was explained by the difference in the Δ*δ*, i.e., the miscibility was highest in the NBR-H/PVC blend with a small Δ*δ* in which a single glass transition was seen, followed by the NBR-M/PVC blend, and it was lowest in the NBR-L/PVC blend, with a large Δ*δ*, in which two separate glass transitions were seen.

### 3.3. Two-Phase Morphology

**Figure 2:**
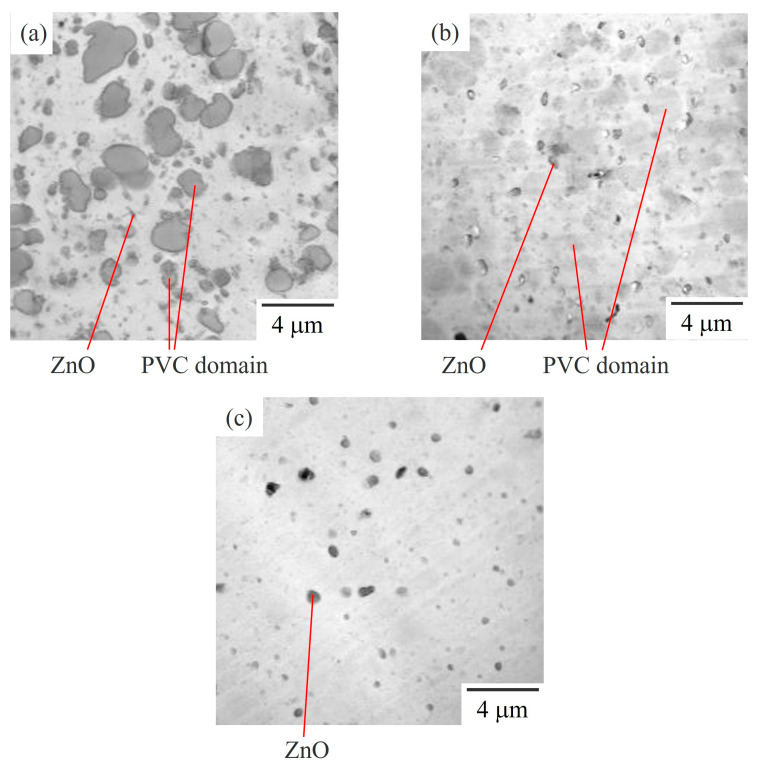
Low-magnification BF-mode STEM images of the 60/40 NBR/PVC blends with a different AN content in the NBR: (**a**) NBR-L/PVC; (**b**) NBR-M/PVC; (**c**) NBR-H/PVC.

Figure 2 shows a series of BF-mode STEM images of 60/40 NBR/PVC with different AN contents in the NBR, which were the same specimens as were used for the DMA measurements in Figure 1. Large dark domains with a diameter of approximately 1 μm were dispersed in the white matrix in the NBR-L/PVC and NBR-M/PVC blends (Figure 2a,b). The contrast between the domain and matrix was much lower in the NBR-M/PVC blend than in the NBR-L/PVC blend. The difference in the contrast is attributed to the difference in the miscibility; the miscibility of the NBR-M/PVC blend is higher than that of the NBR-L/PVC blend, as demonstrated by the DMA results shown in Figure 1. No phase-separated structure was seen in the NBR-H/PVC blend, in which a single glass transition was seen, in Figure 1, but only domains of the vulcanization accelerator, ZnO, were seen in the matrix.

**Figure 3:**
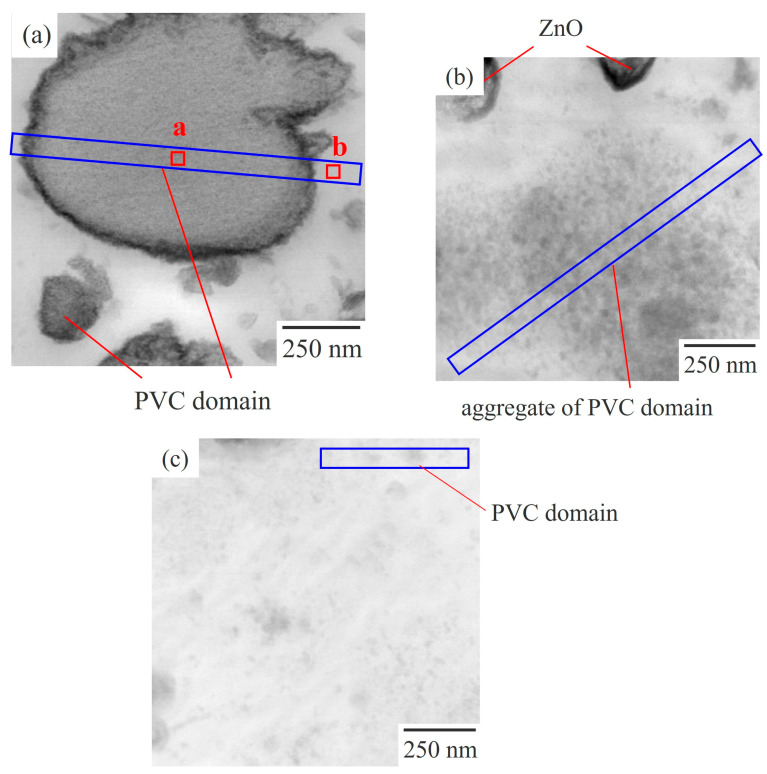
High-magnification BF-mode STEM images of the 60/40 NBR/PVC blends with a different AN content in the NBR: (**a**) NBR-L/PVC; (**b**) NBR-M/PVC; (**c**) NBR-H/PVC.

Figure 3 shows high-magnification BF-mode STEM images of 60/40 NBR/PVC with different AN contents in the NBR, corresponding to the STEM images in Figure 2. Large dark domains with a diameter of approximately 1 μm, and small ones with a diameter of approximately 200 nm were dispersed in the white matrix, and a sharp interface was seen between the domain and the matrix in the NBR-L/PVC blend (Figure 3a). By contrast, such a large domain structure with a sharp interface was not seen, but small domains with diameters of approximately several tens of nanometers were seen, in the NBR-M/PVC and NBR-H/PVC blends (Figure 3b,c). Small domains were aggregated to form large domains of size 1 μm in the NBR-M/PVC blend, whereas they were isolated in the matrix in the NBR-H/PVC blend. Thus, the high-magnification STEM images showed that the large domains in Figure 2b consisted of aggregated small domains, and the existence of a small phase-separated structure, which could not be observed in Figure 2c.

**Figure 4:**
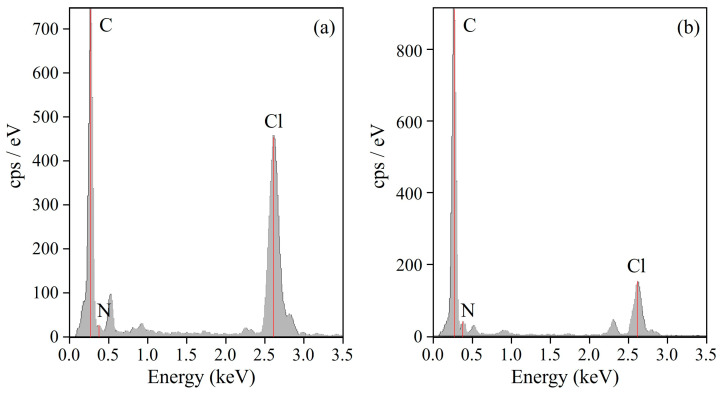
STEM-EDS spectra of the 60/40 NBR-L/PVC blend: (**a**) domain indicated by a in Figure 3a; (**b**) matrix indicated by b in Figure 3a.

Figure 4 shows the EDS spectra at the domain and matrix of 60/40 NBR-L/PVC, indicated by a and b in the STEM image of Figure 3a. Here, the thickness of the ultrathin section of the blend specimen was 100–150 nm, which was thinner than the phase-separated structure. The peaks of the carbon (C)-Kα and nitrogen (N)-Kα from the NBR, and the chlorine (Cl)-Kα from the PVC, were seen at 0.277, 0.392, and 2.621 keV, respectively. The intensity of the Cl spectra was strong in the domain, whereas it was weak in the matrix (Figure 4a). By contrast, the intensity of the N spectra was weak in the domain, whereas it was strong in the matrix (Figure 4b). Hence, the domains are assigned to the PVC phase, and the matrix is assigned to the NBR phase.

**Figure 5:**
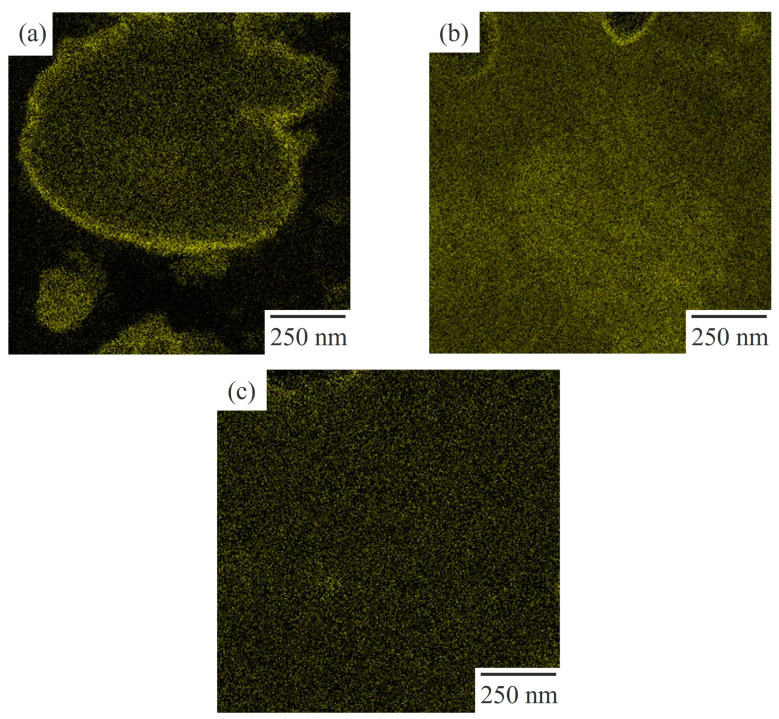
STEM-EDS elemental mapping with chlorine for the 60/40 NBR/PVC blends with a different AN content in the NBR: (**a**) NBR-L/PVC; (**b**) NBR-M/PVC; (**c**) NBR-H/PVC.

Figure 5 shows the STEM micrographs obtained using EDS elemental mapping analysis for the target element of chlorine, using the EDS spectra (Figure 4) for 60/40 NBR/PVC with a different AN content in the NBR, in which the area of the micrographs was the same as that of the STEM image shown in Figure 3. The PVC-rich region with a large amount of PVC is seen as yellow, whereas the NBR-rich region with a small amount of PVC is seen as black. Yellow domains were dispersed in the black matrix in the NBR-L/PVC blend (Figure 5a). Hence, the large dark domain with a diameter of approximately 1 μm, and the small ones shown in Figure 3a, are assigned to the PVC domains, and the white matrix is assigned to the NBR matrix, as described in the EDS spectra shown in Figure 4. As shown in Figure S2, the phase-separated structure of the PVC domain dispersed in the NBR-L matrix could not be identified using optical microscopy, although large PVC domains with a diameter of approximately 1 μm existed in the NBR-L matrix, due to the low contrast of the phase-separated structure, and the low transparency caused by the existence of the additives. Thus, STEM–EDS elemental mapping analysis is helpful in clarifying the phase-separated structure of low-transparency polymer blends that cannot be observed using optical microscopy.

Both the domain and matrix are yellow in the NBR-M/PVC and NBR-H/PVC blends (Figure 5b,c), suggesting that a large amount of PVC exists in both the domain and matrix; i.e., each of the component polymers exists in the partner polymer-rich phase. Large dense yellow domains, with a diameter of approximately 1 μm, are seen in the NBR-M/PVC blend. Thus, the large domain consisting of aggregates of small domains in the NBR-M/PVC blend shown in Figure 3b is assigned to the PVC phase. Note here that there were some domains of the vulcanization accelerator, ZnO, with a size of several hundred nanometers that were seen as dense dark domains in Figure 2b and Figure 3b, and a black matrix in Figure 5b [13]. By contrast, no structure was seen in the NBR-H/PVC blend (Figure 5c), although small domains with a diameter of approximately several tens of nanometers dispersed in the matrix are seen in Figure 3c. No structure in Figure 5c is attributed to the small size of domains, and the small difference in the PVC component between the PVC-rich phase and NBR-rich one, as discussed in the next section.

### 3.4. Concentration Distribution

**Figure 6:**
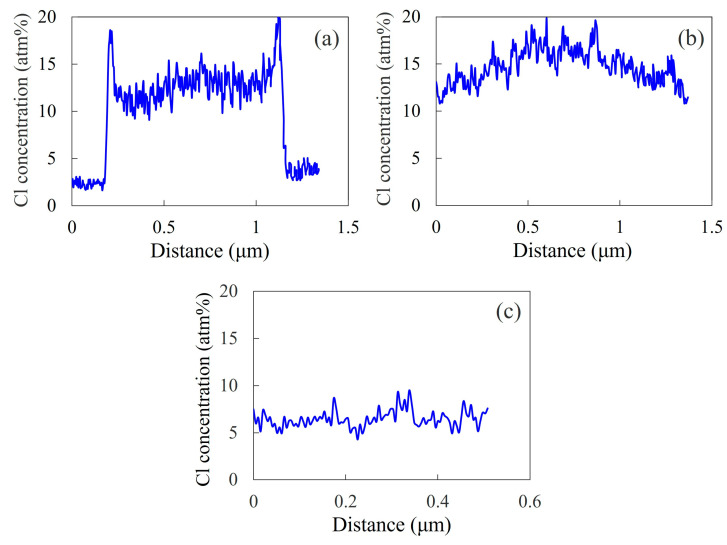
Concentration distribution of chlorine for the 60/40 NBR/PVC blends with a different AN content in the NBR: (**a**) NBR-L/PVC; (**b**) NBR-M/PVC; (**c**) NBR-H/PVC.

As shown in Figure 6, the concentration distribution of the chlorine in PVC could be observed in the 60/40 NBR/PVC blend via STEM–EDS elemental mapping analysis, using dual SDDs, due to the high sensitivity for the chlorine element. Here, the concentration distribution was obtained in the region indicated by a blue rectangle, shown in Figure 3, using the EDS spectra (Figure 4). The concentration distributions obtained at different areas for the NBR-L/PVC, NBR-M/PVC, and NBR-H/PVC blends are shown in Figures S3–S5 as Supplementary material, respectively. The concentration of chlorine is higher in the PVC-rich phase, and it is lower in the NBR-L-rich one. The concentration difference in the PVC between the PVC domain and the NBR matrix was large, and the interface was sharp in the NBR-L/PVC blend (Figure 6a). The interesting result here is that the PVC concentration in the PVC domain was lower than that around the interface, suggesting that the NBR-L is mixed with PVC in the PVC domain. A high PVC concentration around the interface was confirmed in different areas, and different PVC domains (Figure S3). The large PVC concentration around the interface might be attributed to the exclusion of PVC from the NBR-rich matrix, due to the cross-linking of the NBR and the suppression of the diffusion of the excluded PVC into the PVC-rich domain, due to the low mobility of the PVC-rich domain, owing to the high *T*_g_ (Figure 1a). Excluded PVC was also observed around the interface of ZnO, and the NBR-rich matrix, as shown in Figure 5b. Thus, the small shift and small tail in the two separate tan *δ* peaks observed via the DMA analysis shown in Figure 1a are not attributed to the mixing of NBR-L and PVC at the interface, but to the mixing of the component polymers in the PVC domain, although they are usually considered to be attributed to the mixing of the component polymers at the interface [8,9,10,11,12].

The concentration difference in the PVC in the PVC domain and the NBR matrix was small in the NBR-M/PVC and NBR-H/PVC blends (Figure 6b,c). The small concentration difference is attributed to the partial miscibility of NBR and PVC, in which each of the component polymers exists in the partner polymer-rich phase. The NBR-H/PVC blend was not a single-phase miscible blend, but a two-phase blend with a concentration difference, although a blend with a single tan *δ* peak is usually considered to be a single-phase miscible one. The existence of the concentration distribution was confirmed at different areas in the NBR-M/PVC blend (Figure S4), but there were also regions in which a concentration distribution was not detected in the NBR-H/PVC blend, due to the small size of the domains, and the small concentration difference (Figure S5). The concentration difference in the NBR-H/PVC blend was much smaller than that in the NBR-M/PVC blend, due to the difference in the miscibility; i.e., the miscibility of the NBR-H/PVC blend was higher than that of the NBR-M/PVC blend, as suggested by the DMA result in Figure 1, and the solubility parameter difference in Table 2.

Thus, details of the different phase-separated structures of the NBR/PVC blends at different AN contents could be clarified via STEM–EDS elemental mapping analysis, using modern dual SDDs, whereas they were difficult to clarify using only DMA measurement.

### 3.5. Phase-Separated Structure of NBR/PVC Blends

**Figure 7:**
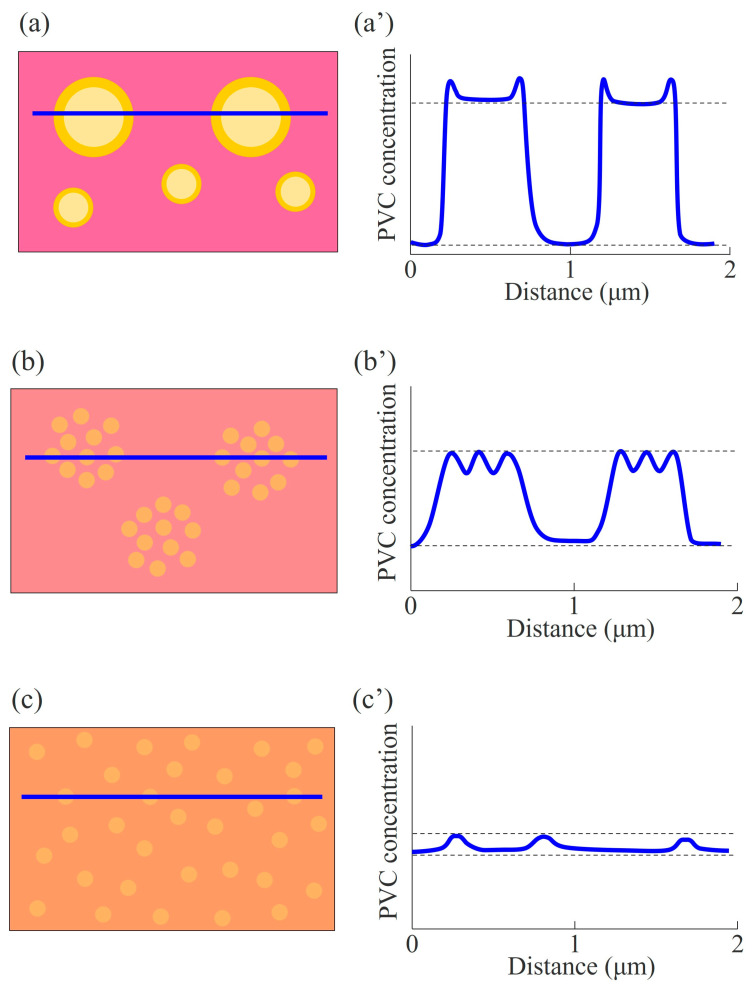
Schematic illustrations for the phase-separated structure and concentration distribution of the 60/40 NBR/PVC at different AN contents: (**a**,**a**’) NBR-L/PVC; (**b**,**b**’) NBR-M/PVC; (**c**,**c**’) NBR-H/PVC.

As expected from the solubility parameter difference, Δ*δ*, the miscibility and phase-separated structure were different depending on the AN content in the NBR in the 60/40 NBR/PVC blends; i.e., the miscibility was highest in the NBR-H/PVC blend, followed by the NBR-M/PVC blend, and it was lowest in the NBR-L/PVC blend. Figure 7 shows schematic illustrations, suggested by the DMA measurements and STEM–EDS mapping analysis, for the phase-separated structure and concentration distribution of the blends with different AN contents. Large and small PVC-rich domains are dispersed in the NBR-L-rich matrix in the NBR-L/PVC blend (Figure 7a). The concentration difference between the PVC-rich domain and the NBR-L-rich matrix is high, but the PVC concentration in the domain is lower than that at the interface, due to the mixing of NBR-L and PVC in the PVC domain. Partially miscible PVC-rich small domains are aggregated to form large domains in the NBR-M-rich matrix in the NBR-M/PVC blend (Figure 7b), whereas partially miscible small PVC-rich domains are isolated in the NBR-H-rich matrix in the NBR-H/PVC blend (Figure 7c). The concentration difference in the PVC in the PVC-rich domain and the NBR-rich matrix was smaller as the AN content was higher, although the blends contained the same PVC content of 40 wt%, due to the increase in the miscibility with increasing AN content. The concentration difference depending on the AN content at the same PVC content might be attributed to the phase-separated structure induced by the liquid–liquid phase separation at the two-phase region in the phase diagram, and the difference in the degree of miscibility depending on the AN content.

As mentioned in the Introduction, a broad tan *δ* peak is desired for the application of damping material. By combining the tan *δ* peaks shown in Figure 1 and the STEM-EDS results shown in Figure 2, Figure 3, Figure 4, Figure 5, Figure 6 and Figure 7, the existence of partially miscible small domains with a size of several tens of nanometers, and the small concentration difference between the domain and the matrix might be required to attain a broad tan *δ* peak.

## 4. Conclusions

We found that different phase-separated structures with different concentration distributions were obtained in NBR/PVC blends at the same blend composition of 60/40, depending on the AN content in the NBR (18.0%, 29.0%, and 33.5%). Two separate sharp tan *δ* peaks were seen in the NBR-L/PVC blend, whereas a broad tan *δ* peak was seen in the NBR-M/PVC and NBR-H/PVC blends. The difference is attributed to the difference in the miscibility, explained by the solubility parameter difference, Δ*δ*; i.e., Δ*δ* decreased due to an increase in the degree of miscibility with the increasing AN content. The STEM–EDS elemental analysis with the target element of chlorine, using dual SDDs, showed, for the phase-separated structure of the blends with a different AN content, that (1) the NBR was mixed into the large PVC domains in the two-phase structure in the NBR-L/PVC blend, although two separate tan *δ* peaks were seen, (2) excluded PVC existed at the interface in the NBR-L/PVC, (3) a two-phase structure was observed in the NBR-H/PVC blend, although a single tan *δ* peak was seen, (4) the concentration difference in the PVC between the PVC domain and the NBR matrix became smaller with the increasing miscibility, as the AN content was higher, although the blends contained the same PVC content of 40 wt%. To our knowledge, this is a first study to clarify the difference in the concentration distribution of the two-phase structure depending on the degree of miscibility. STEM–EDS elemental analysis is a powerful method for clarifying the details of the phase-separated structure, and is also useful in understanding the structure and property of the NBR/PVC nanocomposites, and the recycled blends in which the detail of the phase-separated structure has not been clarified [38,39,40]. The detail in the phase-separated structure is significant for practical applications. For instance, the existence of partially miscible small domains with a size of several tens of nanometers, and the small concentration difference between the domain and matrix observed in the NBR-H/PVC and NBR-M/PBC blends, might be required to attain the broad tan *δ* peak which is desirable in the application of damping materials.

## Figures and Tables

**Table 1:** List of stabilizer and additives mixed into the NBR/PVC blends *^1^.

Material	Grade	Supplier	Additive Amount (g)
stabilizer for PVC	ADK STAB	Adeka Corp., Tokyo, Japan	0.01
	SC-308E		
cross-linking agent	colloidal	Hosoi Chemical Industry Co., Ltd., Tokyo, Japan	0.018
	sulfur		
zinc oxide	JIS-certified	Hakusui Tech Co., Ltd., Osaka, Japan	0.036
(ZnO)	Class 2 *^2^		
stearic acid	stearic acid	NOF Corp., Tokyo, Japan	0.012
N-cyclohexyl-2-	Nocceler CZ	Ouchi Shinko Chemical Industrial Co., Ltd., Tokyo, Japan	0.018
benzothiazolyl			
sulfenamide (CZ)			
tetramethylthiuram disulfide (TT)	Nocceler TT	Ouchi Shinko Chemical Industrial Co., Ltd., Tokyo, Japan	0.002

*^1^ The weights of NBR and PVC for the melt mixing were 0.9 g and 0.6 g, respectively. *^2^ The ZnO was a JIS-certified Class 2 compound, in which the purity was more than 99.5%.

**Table 2:** *E*_C_, *V*, and *δ*_i_ of a series of NBRs and PVCs, and Δ*δ* (= *δ*_PVC_ − *δ*_NBR_) for a series of NBRs.

Polymer	AN (%)	*E*_C_ (J/mol)	*V* (cm^3^/mol)	*δ*_i_ ((MPa)^1/2^)	Δ*δ* (=*δ*_PVC_ − *δ*_NBR_) ((MPa)^1/2^)
NBR-L	18.0	21,272	55.6	19.6	3.0
NBR-M	29.0	22,966	53.4	20.7	1.9
NBR-H	33.5	23,659	52.5	21.2	1.4
PVC	-	19,920	39.1	22.6	-

## Data Availability

Not applicable.

## Supplementary Materials

The following supporting information can be downloaded at: https://www.mdpi.com/article/10.3390/polym15163343/s1, Figure S1: *E*_C_ and *V* of the constituents of NBR and PVC, and the calculated procedure of the solubility parameter; Figure S2: Optical micrograph of the 60/40 NBR-L/PVC, in which PVC domains with a diameter about 1 μm dispersed in the NBR matrix were observed via the STEM images shown in Figure 3a; Figure S3: Concentration distribution of chlorine for the 60/40 NBR-L/PVC at different areas; Figure S4: Concentration distribution of chlorine for the 60/40 NBR-M/PVC at different areas; Figure S5: Concentration distribution of chlorine for the 60/40 NBR-H/PVC at different areas.
