# Supplementary material for: Phase-Separated Structure of NBR/PVC Blends with Different Acrylonitrile Contents Investigated Using STEM–EDS Mapping Analysis
Source: Polymers (Basel). 2023 Aug 9;15(16):3343. doi: 10.3390/polym15163343 (PMC10459754; doi:10.3390/polym15163343)
Supplement: Supplementary file 1 [file polymers-15-03343-s001.zip › polymers-2542592-supplementary.pdf]

Supplementary materials for

Phase-separated structure of NBR/PVC blends with different acrylonitrile contents investigated using STEM-EDS mapping analysis

Yuka Komori*[1, 2] • Aoi Taniguchi[2] • Haruhisa Shibata[1] • Shinya Goto[1] • Hiromu Saito[2]

[1]Materials Engineering R&D Division, DENSO CORPORATION, Kariya-shi 448-8661, Aichi, Japan

[2]Department of Organic and Polymer Materials Chemistry, Tokyo University of Agriculture and Technology, Koganei-shi 184-8588, Tokyo, Japan

*E*c and *V* of the constituents of NBR and PVC

group	cohesive energy E_C (J/mol)	molar volume V (cm^3/mol)
CH_2	4940	16.1
CH	3430	–1.0
– CH =	4310	13.5
CN	25530	24.0
Cl	11550	24.0

Acrylonitrile part of NBR

$E_C = 4940 + 3430 + 25530 = 33900$

$V = 16.1 + (-1.0) + 24.0 = 39.1$

Butadiene part of NBR

$E_C = 4940 + 4940 + 4310 + 4310 = 18500$

$V = 16.1 + 16.1 + 13.5 + 13.5 = 59.2$

NBR-L (AN content: 18.0 wt%)

$E_C = 33900 \times 0.18 + 18500 \times 0.82 = 21272$

$V = 39.1 \times 0.18 + 59.2 \times 0.82 = 55.582$

$\delta_{NBR\text{-}L} = (E_C/V)^{0.5} = 19.6$

NBR-M (AN content: 29.0 wt%)

$E_C = 33900 \times 0.29 + 18500 \times 0.71 = 22966$

$V = 39.1 \times 0.29 + 59.2 \times 0.71 = 53.371$

$\delta_{NBR\text{-}M} = (E_C/V)^{0.5} = 20.7$

NBR-H (AN content: 33.5 wt%)

$E_C = 33900 \times 0.335 + 18500 \times 0.665 = 23659$

$V = 39.1 \times 0.335 + 59.2 \times 0.665 = 52.4665$

$\delta_{NBR\text{-}H} = (E_C/V)^{0.5} = 21.2$

PVC

$E_C = 4940 + 3430 + 11550 = 19920$

$V = 16.1 + (-1.0) + 24.0 = 39.1$

$\delta_{PVC} = (E_C/V)^{0.5} = 22.6$

Figure S1. *E*c and *V* of the constituents of NBR and PVC, and the calculated procedure of the solubility parameter.

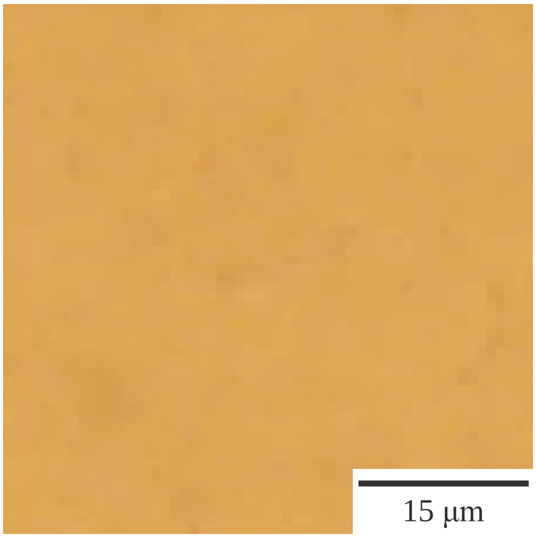

Figure S2. Optical micrograph of the 60/40 NBR-L/PVC, in which PVC domains with a diameter about 1 μm dispersed in the NBR matrix were observed via the STEM images shown in Figure 3a.

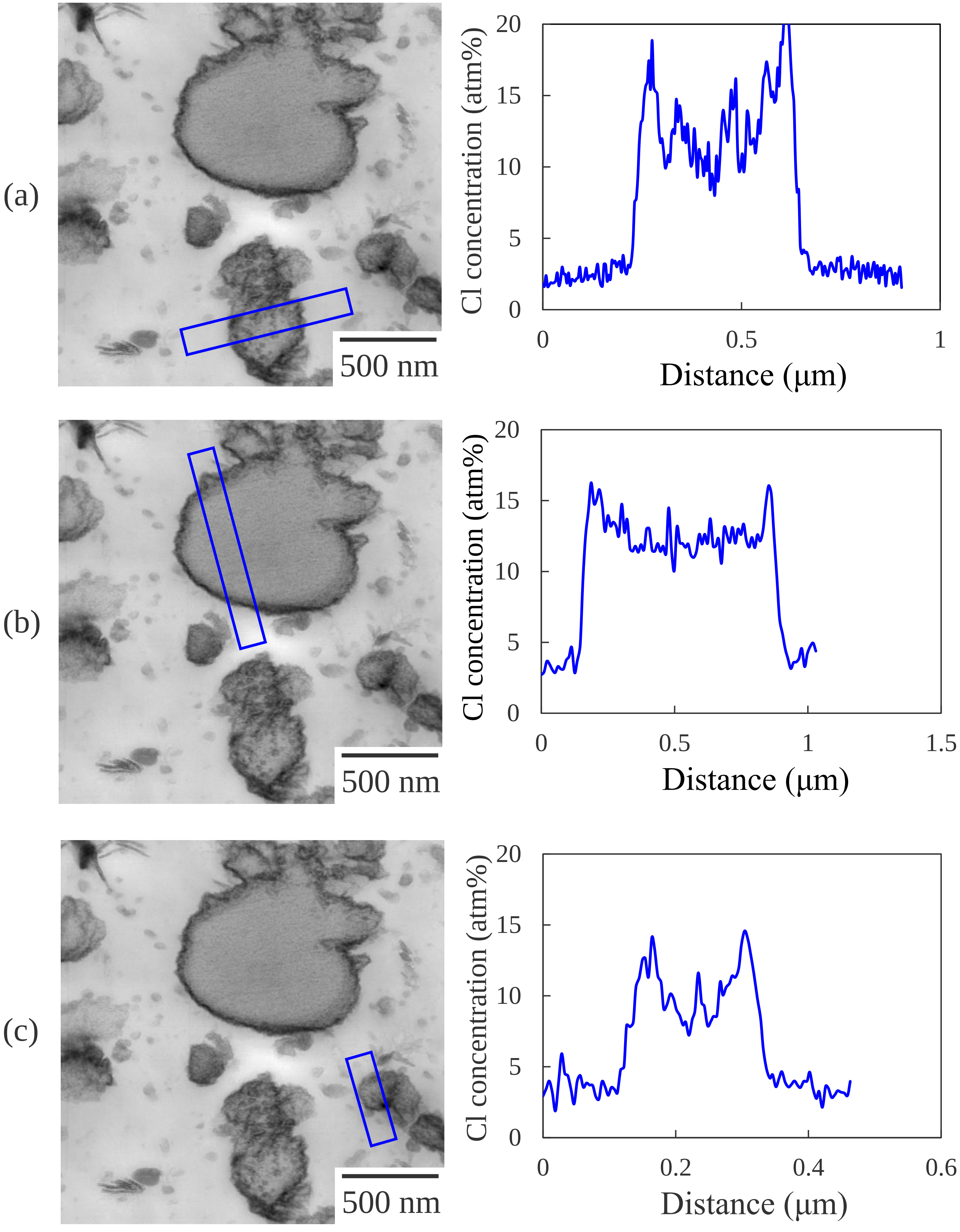

Figure S3. Concentration distribution of chlorine for the 60/40 NBR-L/PVC at different areas.

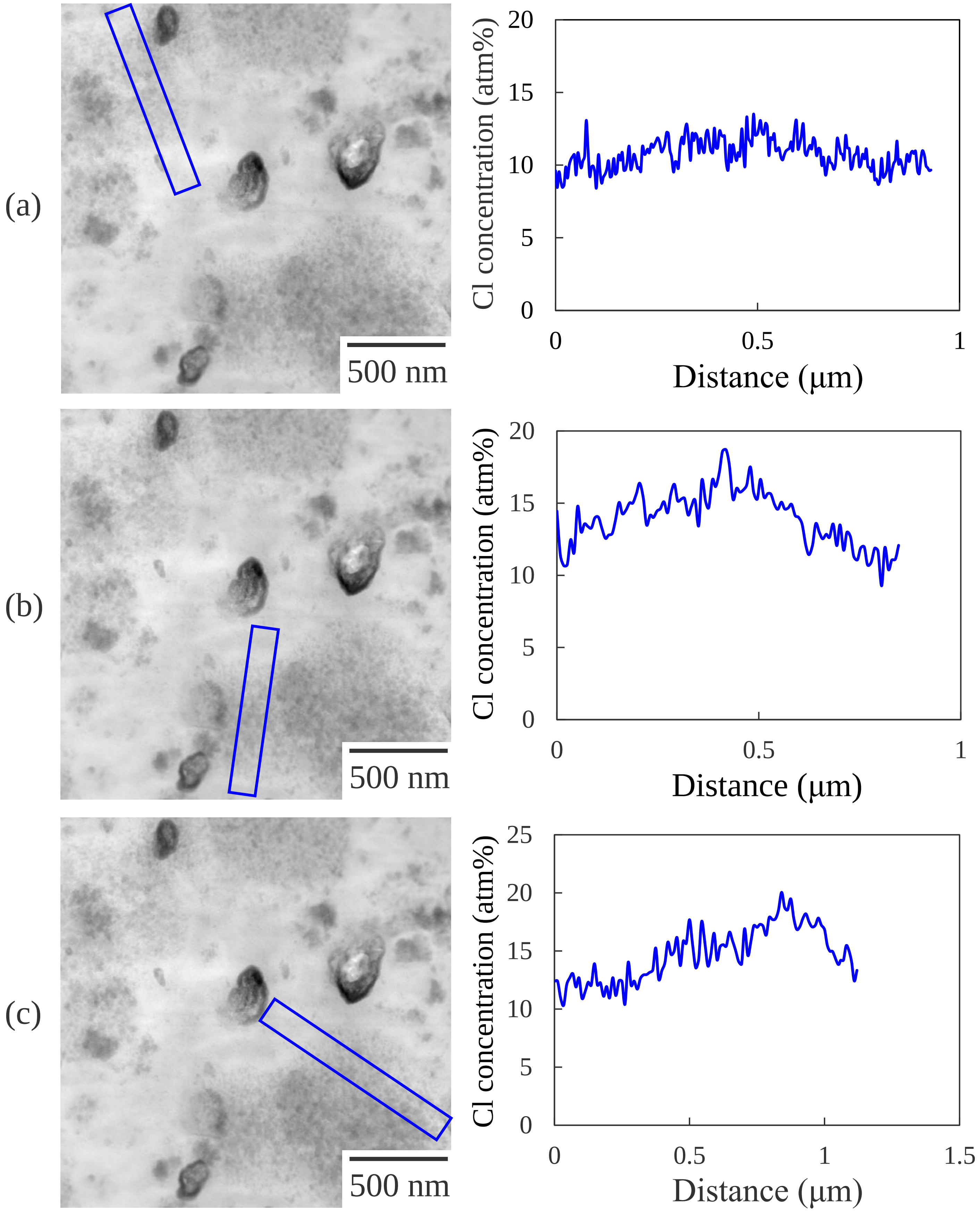

Figure S4. Concentration distribution of chlorine for the 60/40 NBR-M/PVC at different areas.

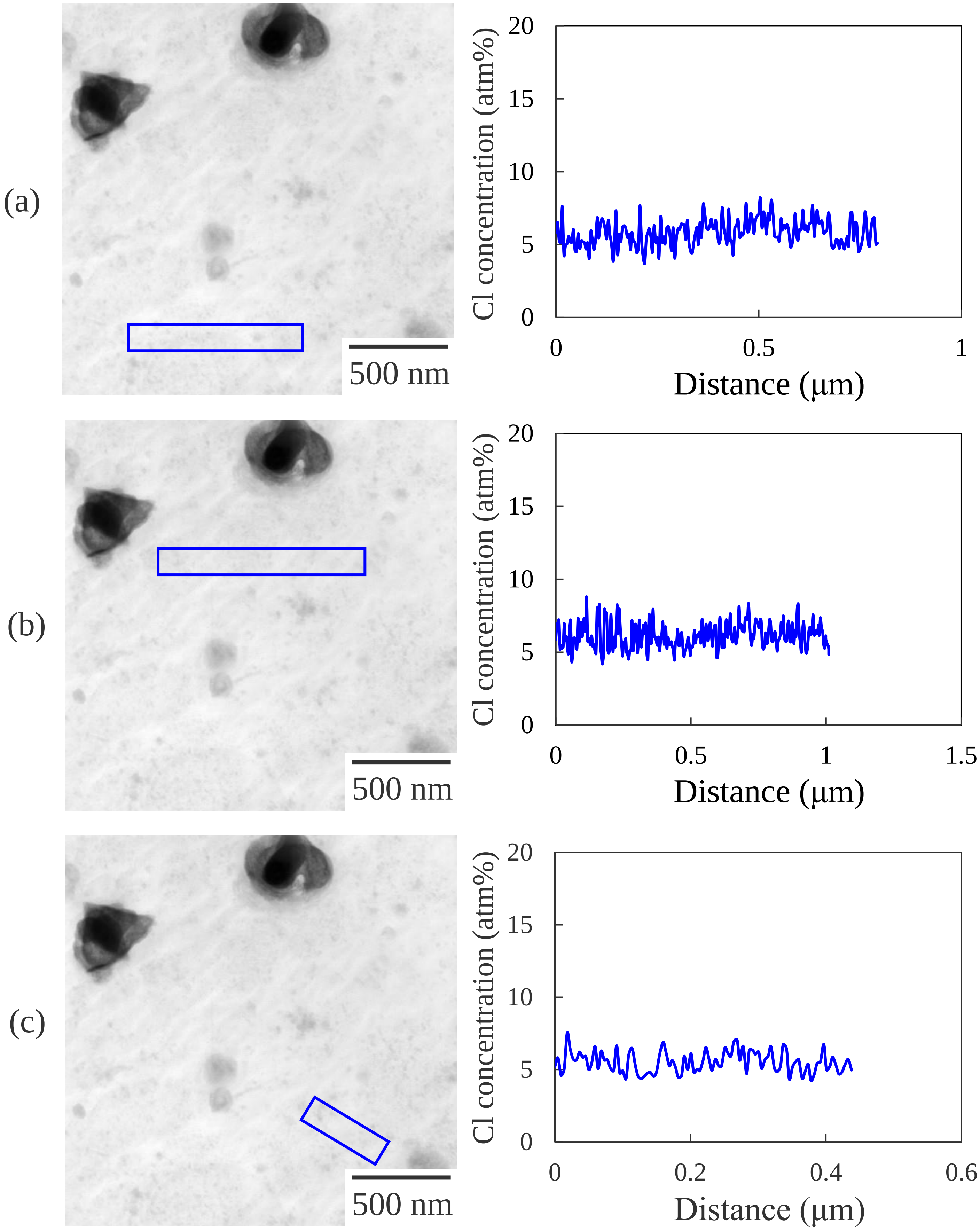

Figure S5. Concentration distribution of chlorine for the 60/40 NBR-H/PVC at different areas.
